# Objective Evaluation of Ocular Surface Adverse Effects in Patients Receiving Topical Antiglaucoma Treatment

**DOI:** 10.22336/rjo.2025.31

**Published:** 2025

**Authors:** Cristina-Mihaela Anghel-Timaru, Daniela Adriana Iliescu, Leon Zăgrean

**Affiliations:** 1 Division of Physiology-Neuroscience - Department of Functional Neurosciences “Carol Davila” University of Medicine and Pharmacy, Bucharest, Romania; 2 Ama Optimex Ophthalmology Clinic, Bucharest, Romania

**Keywords:** glaucoma, topical medication, ocular surface, TBUT, Schirmer, OAG = open-angle glaucoma, IOP = intraocular pressure, RNFL = retinal nerve fibre layer, IOH = intraocular hypertension, OSD = ocular surface disease, TBUT = tear break-up time, IOL = intraocular lens, SPK = superficial punctate keratitis, PL = light perception, RE = right eye, LE = left eye, OS = ocular surface, IU = international unit

## Abstract

Topical treatments remain the primary therapeutic strategy for glaucoma, with the primary objective of reducing intraocular pressure (IOP), the only modifiable risk factor for the disease. While these medications are effective in lowering IOP, they are also associated with a variety of potential adverse effects.

This study aims to compare the effects of various topical antiglaucoma medications on the ocular surface in patients with open-angle glaucoma or intraocular hypertension. Patients were classified based on their treatment type and assessed using the Schirmer test, tear break-up time (TBUT), and fluorescein staining. The monitoring period spanned from February 2019 to September 2024.

Preliminary results suggested that the daily number of drops administered may have a more significant impact on outcomes than the presence or absence of preservatives in the eye drops.

## Introduction

Glaucoma is a group of ocular diseases characterized by progressive optic neuropathy, resulting from the gradual degeneration of retinal nerve fibers [1]. This leads to irreversible visual acuity impairment and a substantial decline in the patient’s quality of life [2]. While various forms of glaucoma exist, each with distinct etiologies and characteristics, this study primarily focuses on primary open-angle glaucoma (OAG) and intraocular hypertension (IOP).

OAG is defined by the presence of elevated intraocular pressure (IOP), exceeding 21 mmHg, in conjunction with alterations in the retinal nerve fiber layer (RNFL) and visual field defects [3]. In contrast, IOH is characterized by elevated IOP without associated changes in the RNFL or visual field, which may or may not present with symptoms [4].

A significant challenge in managing glaucoma lies in determining the most appropriate treatment for each patient. This involves balancing the potential adverse effects with the anticipated benefits, with an emphasis on optimizing therapeutic outcomes. Given the unclear etiology of this condition, treatment is primarily aimed at reducing intraocular pressure (IOP), which remains the only modifiable risk factor [5]. The therapeutic goal is to achieve a target IOP level that slows or halts the progression of glaucoma.

There are four primary classes of topical medications used to manage intraocular pressure (IOP) in glaucoma: alpha agonists, beta blockers, carbonic anhydrase inhibitors, and prostaglandin analogues. Initial treatment typically involves the use of a single-class topical agent. However, in most cases, the combination of two or more topical medications is required to achieve the desired therapeutic target for intraocular pressure (IOP) [6]. To enhance treatment adherence and improve patients’ quality of life, fixed-combination formulations have been developed, which combine two antiglaucoma agents in a single bottle.

While these treatments are crucial for managing glaucoma, their associated adverse effects on the ocular surface have prompted extensive research aimed at mitigating or eliminating these side effects. Given that glaucoma typically necessitates lifelong treatment with topical medications (unless surgical intervention is indicated), patient adherence to the prescribed regimen is of paramount importance. Adherence is essential not only for slowing the progression of the disease but also for improving the patient’s overall quality of life [7,8].

A key factor influencing treatment compliance is the reduction of adverse effects, which, in turn, can enhance patient adherence to the prescribed therapy [9].

A crucial consideration regarding the ocular surface is the coexistence of glaucoma with other ocular dysfunctions, specifically ocular surface disease (OSD) [10,11]. The majority of glaucoma patients are over the age of 60 and often exhibit signs of dry eye syndrome. A critical aspect in evaluating the effects of topical medication on the ocular surface is the ability to differentiate between symptoms that have emerged since the initiation of topical therapy and those that were pre-existing [12].

Currently, there is no clear consensus regarding the etiology of topical medication-induced ocular surface disease. Two primary theories exist: one attributes the cause to the preservative used in the formulation to prevent contamination and enhance penetrability, while the other suggests that the active substance, which is essential for reducing intraocular pressure (IOP), may be responsible [1,3].

In response to these concerns, preservative-free topical medications have been developed and introduced to the market to reduce adverse effects on the ocular surface, while maintaining efficacy in lowering IOP [14-17].

Currently, studies predominantly suggest that damage to the ocular surface is primarily attributed to the preservative, specifically benzalkonium chloride (BAK), or the frequency of daily administration. It is not so much the preservative itself, but rather the extent of its accumulation, that is believed to cause harm [1,2].

## Materials and methods

The present study aims to investigate the changes in both the ocular surface and intraocular pressure (IOP) as a result of topical medication, with or without preservatives, using objective evaluations of parameters such as IOP, retinal nerve fiber layer (RNFL) thickness, corneal epithelial damage, Schirmer test, and tear film break-up time.

The subjects of the study were patients from the Ama Optimex Clinic who were seen and consulted between February 2019 and July 2021, or between February 2024 and September 2024. These patients were either already undergoing treatment for glaucoma or were newly diagnosed with the condition.

Inclusion criteria:

- age over 18 years;
- primitive open-angle glaucoma or intraocular hypertension diagnosis.

Exclusion criteria:

- age under 18 years;
- surgical interventions for glaucoma;
- surgical interventions on the ocular surface (corneal refractive surgery, corneal transplant);
- significant damage to the corneal surface before using antiglaucoma treatment.

For the consultation and investigation of these patients, the following were necessary:

- Biomicroscope with Goldman tonometer attached;
- Topical ocular anesthetic;
- Fluorescein;
- OCT device (ocular coherence tomography);
- Computerized perimeter.

Establishing subject groups:

- group A: prostaglandin analogue treatment with preservative - 1 administration per day;
- group B: treatment with prostaglandin analogue without preservative - 1 administration per day;
- group C: treatment with fixed combination without preservative - 2 administrations per day;
- group D: treatment with fixed combination with preservative - 2 administrations per day;
- group E: fixed combination + prostaglandin analogue without preservative, three administrations per day;
- group F: fixed combination + prostaglandin analogues with preservative, three administrations per day;
- group G: fixed combination + carbonic anhydrase inhibitor or fixed combination + brimonidine - 4 administrations, with preservative.

It is essential to note that patients’ medication regimens were not consistent throughout the treatment course. Due to either adverse effects experienced by the patients or adjustments made by the treating physician based on consultations and investigations, the treatment was modified to maximize comfort and optimize therapeutic efficacy in controlling disease progression. As a result, the same patient might belong to multiple groups, depending on the treatment regimen followed over a specific period.

### Follow-up of patients

Patient follow-up consultations and investigations were conducted at the Ama Optimex and Medicover clinics.

Regarding the medication regimen, prostaglandin analogues were administered once daily, in the evening. In contrast, fixed-combination medications, such as brimonidine and carbonic anhydrase inhibitors, were prescribed twice daily, in the morning and evening (typically at 8:00 a.m. and 8:00 p.m.).

The initial consultation for each patient included in the study comprised:

- patient history: symptoms that led to presentation to the doctor and the diagnosis of OAG or IOH, other coexisting pathologies, eye treatments followed until presentation;
- a detailed biomicroscopic examination of the anterior pole of the eye, with the administration of mydriatics for the evaluation of the posterior pole;
- measurement of intraocular pressure with the Goldman tonometer;
- checking visual acuity at a distance and when reading;
- performing a Schirmer test;
- in case of erosions or corneal damage, staining with fluorescein;
- measurement of tear break-up time (TBUT-tear break-up time);
- testing the visual field with computerized perimetry;
- performing an optical coherence tomography of the optic nerve to evaluate the layer of retinal nerve fibers;
- performing an anterior chamber Pentacam to measure the thickness of the central cornea.

The follow-up consultation included:

- biomicroscopic examination of the anterior and posterior ocular pole;
- IOP measurement using the Goldman tonometer;
- TBUT measurement;
- performing a Schirmer test;
- performing computerized perimetry (3 months in the first year after diagnosis, then 6 months later);
- optical coherence tomography (after 6 months);
- epithelial map (2 weeks after the initiation or change of treatment).

The degree of ocular surface dysfunction was measured objectively using the tear film breakup time (TBUT) and the Schirmer test.

For the TBUT and Schirmer tests, the degree of ocular dysfunction can be classified as normal (over 10), mild (7-9), moderate (5-7), or severe (<5) [1,8].

Among the patients in the groups, some had diabetes. The present study aimed to investigate the differences between diabetic and non-diabetic individuals, to determine whether diabetes might be a contributing factor in influencing the side effects of treatment.

## Results

The patients included in the study ranged in age from 23 to 83 years; 41 patients were female, and seven were male.

From the total number of patients, 34 patients (70.83%) were diagnosed with open-angle glaucoma, and 14 patients (29.16%) with intraocular hypertension.

From the first presentation to the last documented, the visual acuity of the patients remained the same in 58 eyes, decreased in 25 eyes, improved in 8 eyes, and two eyes, the ability to perceive light was lost.

The improvement in visual acuity was attributed to the replacement of the opacified lens (senile cataract) with an artificial intraocular lens (IOL) following phacoemulsification and implantation of a posterior chamber IOL.

**Fig. 1:**
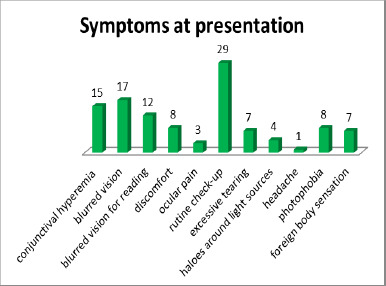
Symptoms that brought patients to consultation leading to diagnosis of the pathology (OAG/IOH)

The reasons for presenting to the ophthalmology consultation, which ultimately led to the diagnosis of the condition, varied among patients (**Fig. 1**).

**Fig. 2:**
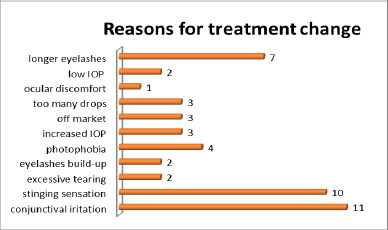
Reasons for changing the initial treatment

**Fig. 3:**
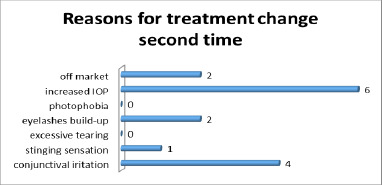
Reasons for changing the treatment for the second time

Due to adverse effects or at the physician’s discretion, the treatment was modified for 14 patients (26 eyes). In 7 patients (12 eyes), the treatment was changed twice (**Fig. 2,3**). Consequently, these patients were classified into multiple groups based on the treatment regimen used.

**Fig. 4:**
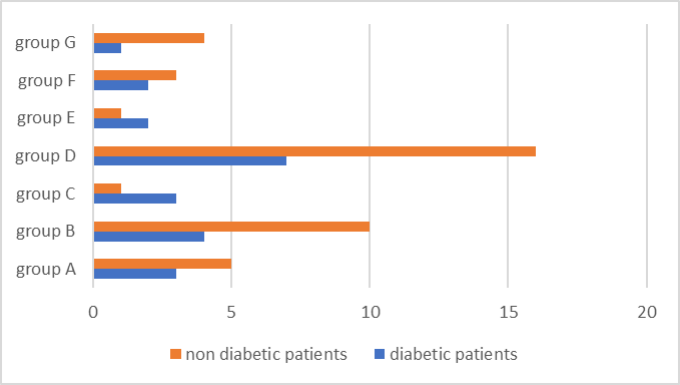
Diabetic and non-diabetic patients

The following figure illustrates the number of patients in each group, distinguishing between diabetic and non-diabetic patients (**Fig. 4**).

Next, the data obtained for each patient group was analyzed.

### Group A

Group A consisted of 8 patients (7 females, one male; 16 eyes), aged between 30 and 77 years, all of whom received topical prostaglandin analogues medication containing a preservative, administered at a dosage of 1 drop per day in the evening.

Among the patients in this group, two were diagnosed with ocular hypertension (IOH), and 6 with open-angle glaucoma (OAG). The visual acuity of the patients at presentation ranged from 0.6 to 1.0 (with 1.0 representing 100% visual acuity).

Of the 16 eyes in Group A, 13 maintained their baseline visual acuity, two experienced a decrease in visual acuity, and one showed an improvement in visual acuity.

At diagnosis, intraocular pressure (IOP) ranged from 21 to 29 mmHg. During treatment, IOP was successfully normalized, with values ranging from 12 to 16 mmHg.

In Group A, the Tear Break-Up Time (TBUT) at baseline ranged from 9 to 13 seconds, and the Schirmer test results ranged from 8 to 14 millimeters. During treatment, TBUT remained stable in two eyes, while it decreased by 1-3 units in the remaining 14 eyes. Similarly, the Schirmer test remained constant for two eyes and decreased by 1-4 units in 14 eyes.

The grading for both the Tear Breakup Time (TBUT) and Schirmer test showed variability throughout the treatment period.

**Fig. 5:**
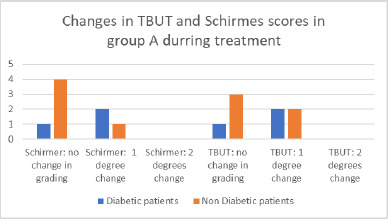
Changes in TBUT and Schirmer test in group A

Regarding the Schirmer test, five patients maintained their initial grading, including one diabetic patient. Three patients experienced a one-grade decrease in their results. No patients demonstrated more significant changes. As illustrated in **Fig. 8**, the majority of patients who showed no change in grading were non-diabetic. Among those who experienced a decrease in grading, two out of three were diabetic (**Fig. 5**).

**Fig. 6:**
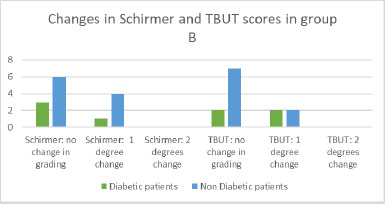
Changes in TBUT and Schirmer test in group B

**Fig. 7:**
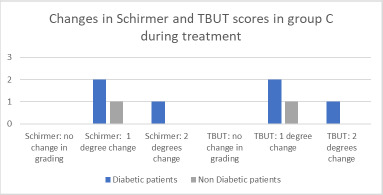
Changes in Schirmer test and TBUT in group C

**Fig. 8:**
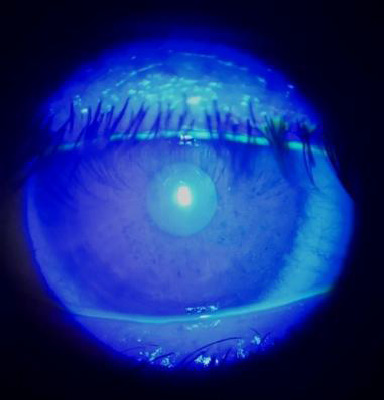
SPK in patient group C

For the TBUT, four patients showed no change in grading, while four others exhibited a one-grade decrease in grading. Similar to the Schirmer test, the majority of patients with stable grades were non-diabetic (**Fig. 5**).

Throughout the treatment, no documented corneal erosions or minor epithelial defects (superficial punctate keratitis, SPK) were observed.

### Group B

Group B consisted of 14 patients (11 females, three males; 28 eyes), aged between 35 and 79 years, who were undergoing treatment with preservative-free prostaglandin analogues administered topically at a dosage of one drop per day in the evening.

Within this cohort, seven patients have been diagnosed with intraocular hypertension (IOH), and 7 with open-angle glaucoma (OAG). Upon presentation, the visual acuity of the patients ranged from 0.7 to 1.0. Of the 28 eyes in Group B, 19 maintained stable visual acuity compared to the initial clinic presentation, one eye showed improvement in visual acuity, and eight eyes experienced a decline in visual acuity.

Regarding intraocular pressure (IOP), in Group B, values at diagnosis ranged from 27 to 59 mmHg. During treatment, IOP decreased, varying between 11 and 24 mmHg.

Tear break-up time (TBUT) at baseline ranged from 10 to 13 seconds, while the Schirmer test values ranged from 10 to 15 millimeters. During treatment, TBUT remained stable in one eye, increased by 1 second in two eyes, and decreased by 1-4 seconds in 25 eyes. Nine patients showed no change in grading, while four others exhibited a one-grade decrease. The majority of patients with stable grades were non-diabetic (**Fig. 6**).

Similarly, the Schirmer test remained unchanged for two eyes, increased by 1 mm in one eye, and decreased by 1-5 millimeters in 25 eyes. Nine patients maintained their initial grading, including three diabetic patients. Five patients experienced a one-grade decrease in their results. No patients demonstrated more significant changes. As illustrated in **Fig. 10**, the majority of patients who showed no change in grading were non-diabetic. Among those who experienced a decrease in grading, one out of five was diabetic (**Fig. 6**).

**Fig. 9:**
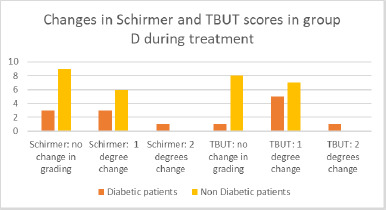
Changes in Schirmer and TBUT in group D

**Fig. 10:**
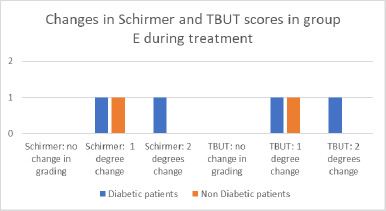
Changes in Schirmer and TBUT in group E

Similar to Group A, no corneal erosions or epithelial defects (e.g., superficial punctate keratitis, SPK) were observed in Group B throughout the treatment period.

### Group C

Group C included four female patients (8 eyes), aged between 50 and 74 years, who were undergoing treatment with a preservative-free fixed combination topical medication, administered at a dosage of one drop twice daily, in the morning and evening.

Among the patients, one was diagnosed with intraocular hypertension (IOH), and three were diagnosed with open-angle glaucoma (OAG). Visual acuity at presentation ranged from 0.2 to 1.0. Of the eight eyes in Group C, 7 maintained stable visual acuity from the initial clinic visit, while one eye exhibited improved visual acuity. Visual acuity measurements were assessed using standard chart testing, with a focus on central vision.

At presentation, intraocular pressure (IOP) in Group C ranged from 21 to 28 mmHg. During treatment, IOP decreased to a range of 12 to 14 mmHg.

In Group C, baseline Tear Break-Up Time (TBUT) values ranged from 9 to 13 seconds, while Schirmer test values ranged from 9 to 14 millimeters. During treatment, TBUT decreased by 3 to 5 seconds in all eight eyes, and Schirmer test values decreased by 2 to 7 millimeters across all eyes. All patients experienced changes in grading for both the Schirmer test and TBUT. Specifically, three patients exhibited a one-grade decrease, while one patient experienced a two-grade reduction. The majority of patients with stable grades were non-diabetic (**Fig. 7**).

In group C, only one patient required a change in treatment due to discomfort caused by the topical application.

There was a documented case of epithelial defects (superficial punctate keratitis - SPK) (**Fig. 8**).

### Group D

Group D consisted of 23 patients (20 females, three males; 44 eyes), aged between 43 and 83 years, who were undergoing treatment with a preservative-containing fixed combination topical medication, administered at a dosage of one drop twice daily, in the morning and evening.

Among the patients, five were diagnosed with intraocular hypertension (IOH), and 18 with open-angle glaucoma (OAG). At presentation, the patients’ visual acuity ranged from uncertain light perception (PL) to 1.0 (representing 100% vision). Of the 44 eyes in Group D, 28 maintained their visual acuity from the initial clinic visit, eight eyes exhibited a decrease in acuity, five eyes showed improvement, and three eyes experienced complete loss of vision. It is important to note that these assessments refer to central visual acuity.

At diagnosis, intraocular pressure (IOP) ranged from 21 to 42 mmHg. During treatment, IOP decreased to a range of 12 to 14 mmHg.

In Group D, the tear break-up time (TBUT) at baseline ranged from 9 to 14 seconds, while the Schirmer test values ranged from 8 to 19 millimeters. During treatment, TBUT remained stable for three eyes, increased by 1 second in 1 eye, and decreased by 1 to 7 seconds in 39 eyes. The Schirmer test remained unchanged for two eyes, increased by 1 to 2 millimeters in 2 eyes, and decreased by 1 to 7 millimeters in 39 eyes.

Most patients who exhibited no change in grade, for both TBUT and Schirmer tests, were non-diabetic, whereas the only patient who experienced a two-grade change in both cases had diabetes. Regarding the one-grade change, no significant differences were observed (**Fig. 9**).

### Group E

Group E consisted of 3 patients (2 females and one male; 6 eyes), aged between 45 and 77 years, who were undergoing treatment with a fixed combination of preservative-containing medication and preservative-free prostaglandin analogues. The treatment regimen included one drop twice daily in the morning and evening, along with one drop of preservative-free prostaglandin analogues in the evening.

All three patients in this group were diagnosed with open-angle glaucoma (OAG). At presentation, their visual acuity ranged from 0.5 to 1.0. Among the six eyes in Group E, 3 maintained their visual acuity from the initial clinic visit, one eye showed a decrease in acuity, and two eyes exhibited improved visual acuity.

At diagnosis, intraocular pressure (IOP) ranged from 24 to 42 mmHg. During treatment, IOP decreased to a range of 11 to 16 mmHg.

In Group E, the baseline tear break-up time (TBUT) ranged from 9 to 10 seconds, while Schirmer test values ranged from 7 to 10 millimeters. During treatment, TBUT remained stable in one eye and decreased by 1 to 2 seconds in five eyes. The Schirmer test decreased by 1 to 2 millimeters in five eyes, while it remained unchanged in one eye. All patients demonstrated modified grading in both the Schirmer test and TBUT. No significant differences were observed between diabetic and non-diabetic patients who showed a one-grade change. The only patient who exhibited a two-grade change in both tests had diabetes (**Fig. 10**).

### Group F

Group F consisted of 5 patients (4 females, one male; 8 eyes), aged between 56 and 82 years, undergoing treatment with a fixed combination of preservative-containing medication and preservative-containing prostaglandin analogues. The treatment regimen included one drop twice daily in the morning and evening, along with one drop in the evening of the preservative-containing prostaglandin analogues.

All five patients in this group were diagnosed with open-angle glaucoma (OAG). At presentation, the patients’ visual acuity ranged from 0.2 to 1.0. Among the 10 eyes, 2 maintained their visual acuity from the initial clinic visit, five eyes exhibited a decrease in acuity, and one eye experienced complete vision loss.

**Fig. 11:**
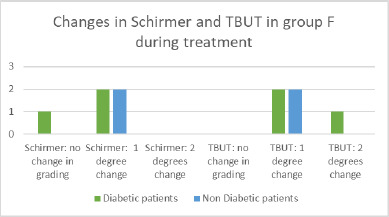
Changes in Schirmer and TBUT in group F

In Group F, the tear break-up time (TBUT) at baseline ranged from 9 to 12 seconds, and the Schirmer test values ranged from 9 to 14 millimeters. During treatment, TBUT decreased by 1 to 6 seconds in all eyes. The Schirmer test decreased by 1 to 7 millimeters in all eyes in Group F. Most patients presented with a one-grade change in both Schirmer test and TBUT, with no significant differences between diabetic and non-diabetic patients (**Fig. 11**).

### Group G

Group G consisted of 5 patients (4 females, one male; 10 eyes), aged between 59 and 82 years, who underwent treatment with a fixed combination of topical medication and another preservative-containing medication, administered at a dosage of one drop twice daily in the morning and evening for both medicines.

All patients in this group were diagnosed with open-angle glaucoma (OAG). At presentation, visual acuity ranged from 0.2 to 1.0. Visual acuity decreased in 9 eyes, while one eye maintained the same acuity.

At diagnosis, intraocular pressure (IOP) in Group G ranged from 31 to 42 mmHg. During treatment, IOP decreased to a range of 10 to 15 mmHg.

**Fig. 12:**
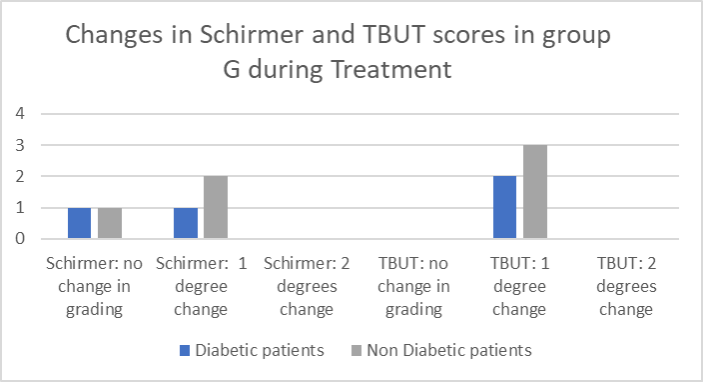
Changes in Schirmer and TBUT in group G

In Group G, the tear break-up time (TBUT) at baseline ranged from 9 to 14 seconds, while the Schirmer test values ranged from 9 to 16 millimeters. During treatment, TBUT decreased by 2 to 7 seconds. The Schirmer test decreased by 1 to 7 millimeters in all eyes of Group G. The Schirmer test grading remained unchanged for two patients and showed a one-degree change in three patients, with no differences observed between diabetic and non-diabetic patients. TBUT showed a one-degree shift in score for all patients (**Fig. 12**).

In Group G, no corneal erosions or notable changes to the ocular surface were observed upon fluorescein staining.

## Results

From an objective standpoint, a more significant reduction in tear break-up time (TBUT) was observed in Groups F and G, which were the only groups to experience a decrease of 7 seconds or more below baseline TBUT. Notably, no patient in these groups maintained the same TBUT as at the start of treatment. In contrast, TBUT values in Groups A and B showed similar trends. When comparing Group C to Group D, a higher proportion of patients in Group C exhibited more pronounced changes in TBUT. The frequency of TBUT changes is listed in **Table 1**.

**Table 1:** TBUT in all groups during treatment

	TBUT down by 1-2 s	TBUT down by 3-4	TBUT down by 5-6 s	TBUT down by >7 s	TBUT the same or better
Group A	64,28%	14,28%	0,00%	0,00%	21,42%
Group B	64,28%	25%	0,00%	0,00%	10,71%
Group C	0,00%	75%	25%	0,00%	0,00%
Group D	55,81%	16,27%	2,32%	0,00%	25,58%
Group E	82,33%	0,00%	0,00%	0,00%	16,66%
Group F	62,50%	25,00%	12,50%	8,33%	0,00%
Group G	0,00%	83,33%	0,00%	16,66%	0,00%

**Fig. 13:**
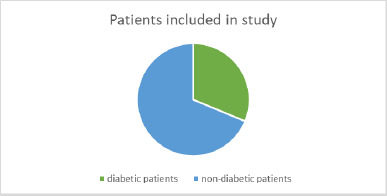
Distribution of patients in the study based on their diabetic status

To more effectively differentiate between diabetic and non-diabetic patients and identify any potential differences in symptoms or objective test results, we analyzed patients based solely on their diabetic status, without considering the group to which they belonged. The study included 15 diabetic patients and 33 non-diabetic patients (**Fig. 13**).

**Fig. 14:**
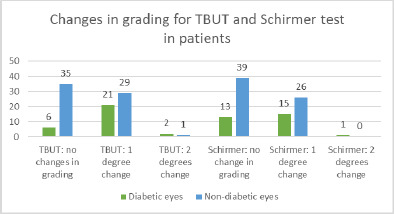
Changes in Schirmer and TBUT tests

**Fig. 15:**
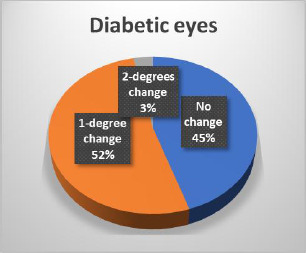
Changes in the Schirmer test in diabetic patients

**Fig. 16:**
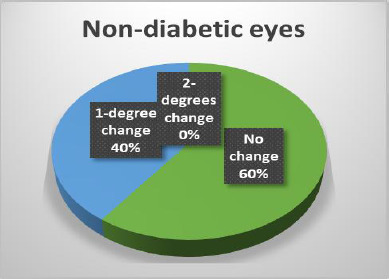
Changes in the Schirmer test in non-diabetic patients

**Fig. 17:**
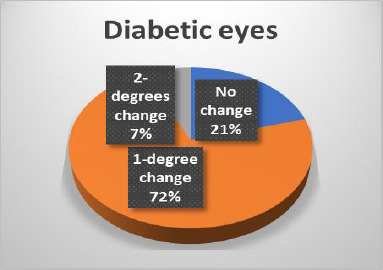
Changes in TBUT in diabetic eyes

**Fig. 18:**
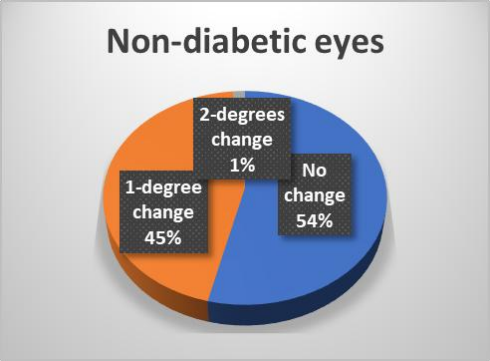
Changes in TBUT in non-diabetic eyes

The results of the Schirmer test indicated a greater susceptibility of diabetic eyes to alterations compared to non-diabetic eyes, with significant changes observed only in diabetic patients (**Fig. 14-16**). In terms of Tear Breakup Time (TBUT), diabetic patients exhibited more pronounced changes than their non-diabetic counterparts (**Fig. 17,18**).

## Discussion

The impact of topical anti-glaucomatous therapy on the ocular surface remains a significant global concern. Numerous studies have investigated this issue, and ongoing efforts are focused on identifying solutions to mitigate these effects and enhance patients’ quality of life.

Several studies suggest that preservative-free topical medications may help reduce ocular surface damage and improve the overall quality of life for patients.

The present study aimed to assess the short- and medium-term effects of anti-glaucomatous medications on the eyes of patients diagnosed with open-angle glaucoma (OAG) or ocular hypertension (IOH), who were receiving topical anti-glaucomatous therapy.

Upon evaluating the seven groups treated with anti-glaucomatous medication, it was evident that groups with multiple daily topical administrations, whether with or without preservatives, exhibited a higher frequency of identifiable adverse effects. Interestingly, the group receiving two daily administrations of preservative-free medication displayed significant ocular damage, in some cases even more pronounced than those receiving three or four drops daily with preservatives.

Regarding diabetic and non-diabetic patients, the findings of the present study suggested that the type of topical treatment had a greater influence on symptomatology than the underlying pathology itself. Objective tests (Schirmer and TBUT) demonstrated more pronounced changes in diabetic patients.

However, the results of this study cannot be generalized due to the small sample size and the unequal distribution of subjects across the groups. Additionally, during the study, other types of topical treatments, such as hyaluronic acid-based artificial tears, topical anti-inflammatory agents, and topical antibiotics, were used to alleviate these adverse effects and improve patients’ quality of life.

The next step is to expand the sample size in each group and standardize the methodology to achieve more robust and conclusive findings.

## Conclusion

The conclusion that can be drawn from these results is that it is not the preservative itself that directly causes adverse effects and ocular surface dysfunction, but rather its cumulative impact due to the administration of a higher number of drops, in conjunction with the active medication.
